# Data on the detection of clinically significant prostate cancer by magnetic resonance imaging (MRI)-guided targeted and systematic biopsy

**DOI:** 10.1016/j.dib.2022.108683

**Published:** 2022-10-19

**Authors:** M Klingebiel, C Arsov, T Ullrich, M Quentin, R Al-Monajjed, D Mally, LM Sawicki, A Hiester, I Esposito, P Albers, G Antoch, L Schimmöller

**Affiliations:** a University Dusseldorf, Medical Faculty, Department of Diagnostic and Interventional Radiology, D-40225 Dusseldorf, Germany; b University Dusseldorf, Medical Faculty, Department of Urology, D-40225 Dusseldorf, Germany; c University Dusseldorf, Medical Faculty, Department of Pathology, D-40225 Dusseldorf, Germany

**Keywords:** Prostatic neoplasms, Multiparametric magnetic resonance imaging, Imaging guided biopsy, Pathology

## Abstract

This is a data article from the original publication “Reasons for missing clinically significant prostate cancer by targeted magnetic resonance imaging/ultrasound fusion-guided biopsy” [1]. From January 2014 to April 2019 a sample collective of 785 patients with 3T multiparametric magnetic resonance imaging (mp-MRI) of the prostate and subsequent combined systematic biopsy (SB) and magnetic resonance imaging/ultrasound (US) fusion-guided biopsy (TB) was retrospectively analyzed. Prostate cancer (PCa) detection by TB and/or additional SB was analyzed.

**Specifications Table**

**Table:** 

Subject:	Radiography and radiology
Specific subject area:	Imaging-guided prostate cancer detection
Type of data:	Table (as .xls file)
How data were acquired:	Clinical assessments: medical reports, lab analysis MRI scans: 3T MRI scanner (Magnetom Trio TIM, Skyra, and Prisma; Siemens Healthcare GmbH, Germany) with an 18-channel phased-array surface coil combined with 32-channel spine coil or a 60-channel phased-array surface coil; DynaCAD software (Version 3 or 4, Philips Healthcare, Invivo Corporation, USA) MRI/US-guided biopsy: UroNAV biopsy system (Philips Healthcare, Invivo Corporation, USA) including 12-core systematic biopsy. Histopathology: histopathological evaluation of biopsy cores and classification according International Society of Urological Pathology (ISUP)
Data format:	Analyzed
Parameters for data collection:	MRI scans: detailed mp-MRI parameters are shown in **Table 1**.
Description of data collection:	Consecutive patients with elevated prostate specific antigen (PSA; measured in ng/ml) levels recruited at the Department of Urology and referred to the Department of Diagnostic and Interventional Radiology for mp-MRI at the Medical Faculty of the University Hospital of Duesseldorf.
Data source location:	University Dusseldorf, Medical Faculty Duesseldorf, Department of Diagnostic and Interventional Radiology, Duesseldorf, Germany
Data accessibility:	The dataset used in this study is available on an open access repository (Zenodo); DOI: 10.5281/zenodo.6834906; https://zenodo.org/record/6834906#.YtFjKDdBwuU
Related research article:	Klingebiel M., Arsov C., Ullrich T., Quentin M., Al-Monajjed R., Mally D., Sawicki L.M., Hiester A., Esposito I., Albers P., Antoch G., Schimmöller L., Reasons for missing clinically significant prostate cancer by targeted magnetic resonance imaging/ultrasound fusion-guided biopsy, Eur J Radiol 2021; 137:109587. DOI: 10.1016/j.ejrad.2021.109587

## Value of the Data

• These data show the detection rates and accuracy of the Prostate Imaging and Reporting Data System (PI-RADS) version 2.1 classification, which is a standardized scoring system for prostate MRI assessment. As standardization in interpretation of prostate MRI is necessary, the presented data confirm that the PI-RADS v2.1 is an excellent tool for csPCa risk stratification.
• The benefit of systematic biopsy (SB) additional to MRI-targeted biopsy (TB) was analysed. As there is an on-going discussion regarding the best biopsy approach, our research suggests that additional SB adds overall a limited value.
• Researchers in radiology and urology may profit from these data because the data can be used for further studies on mpMRI and MRI-guided biopsy (e.g., sample size calculation). Radiologists and urologists can use the results for their prostate biopsy or MRI follow-up decision.

## 1 Data Description

**Fig. 1:**
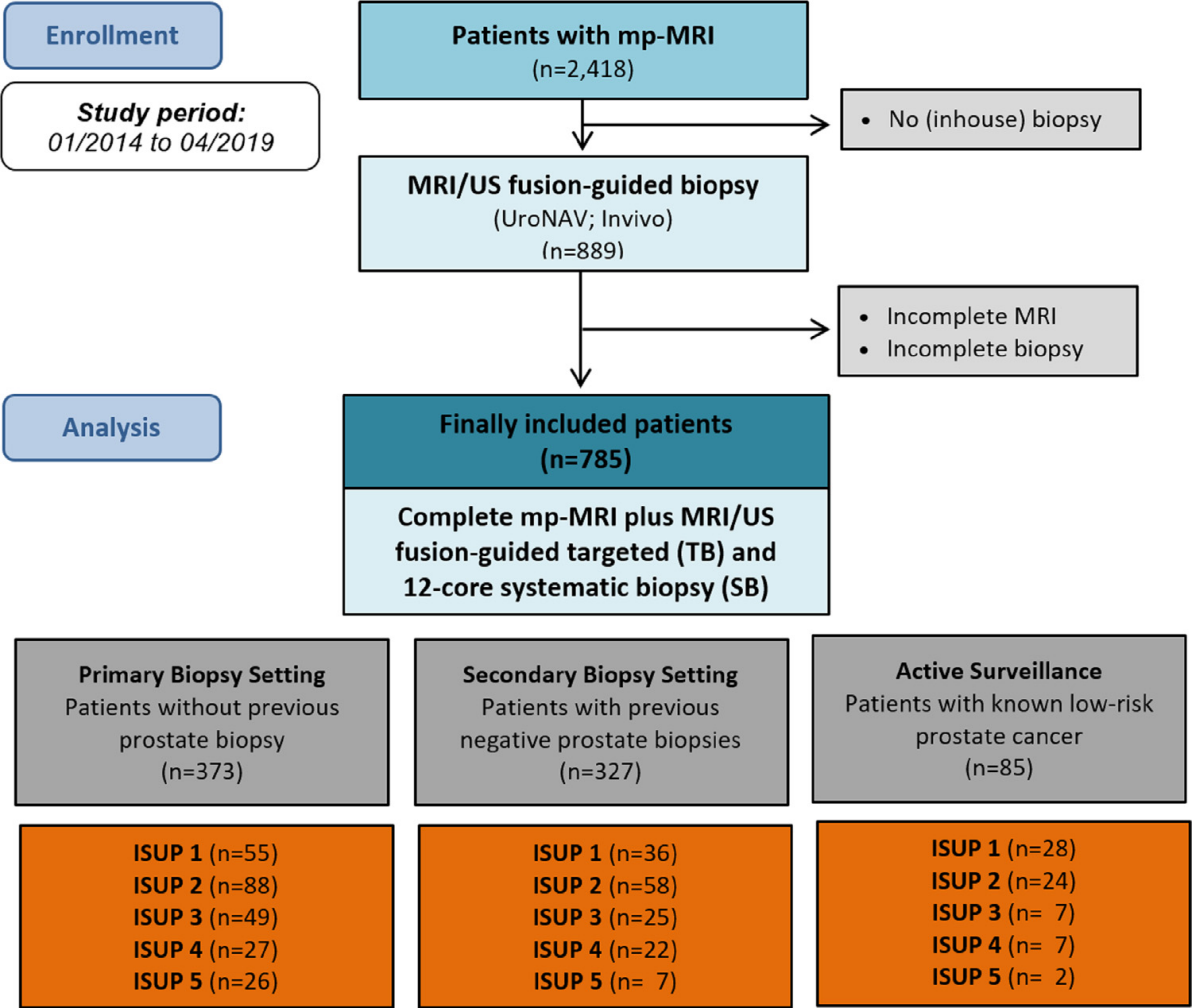
Flowchart.

The presented dataset supports the research article “Reasons for missing clinically significant prostate cancer by targeted magnetic resonance imaging/ultrasound fusion-guided biopsy” [1]. Of 2,418 patients referred to the Departement of Interventional and Diagnostic Radiology (at the University Hospital Duesseldorf, Germany) for mp-MRI of the prostate within the study period, 785 patients (mean age 65 ± 9 years; median PSA value 8.1 ng/ml, interquartile range 5.9 - 12 ng/ml; median prostate volume 47 ml, interquartile range 33 - 66 ml) received complete, subsequent MRI/US fuison-guided targeted (TB) and systematic (SB) biopsy and were finally analysed (Fig. 1). Different 3T MRI scanners were used (Magnetom Trio TIM: n=549, Skyra: n=90, Prisma: n=104, Siemens Healthcare GmbH, Germany; other: n=42) with an 18-channel phased-array surface coil combined with a 32-channel spine coil (n=681) or a 60-channel phased-array surface coil with 30 elements anterior and posterior (n=104) in the supine position. The detailed mp-MRI protocol is shown in Table 1. All mp-MRI were evaluated according to the Prostate Imaging and Reporting Data System (PI-RADS) version 2. PI-RADS is a standardized scoring system to interpret and judge findings in prostate MRI on a scale from 1 (highly likely benign) to 5 (highly likely suspect for a clinically significant prostate cancer; csPCa). Localization and maximum diameter of the MRI index lesion (IL) were documented. PSA values, prostate volume, and PSA density (PSA-D) are provided in the corresponding research article [1]. The overall PCa detection rate was 59% (n=461) including 84% csPCa (n=342; ISUP grade group ≥2). The detection rate of csPCa was the highest in patients with primary biopsy (51%) compared to patients with a secondary biopsy (34%), or patients on Active Surveillance (AS) (47%) (Table 2). Among all patients, TB detected 300 csPCa (88%), whereas SB detected only 247 csPCA (72%) and more non significant (ns) PCa (ISUP grade group 1) (Table 3). In 30 cases (8.8%) a csPCa was detected by SB only and in 67 (20%) cases by TB only. In 12 cases (3.5%) SB lead to a Gleason upgrade ≥ ISUP 2, TB respectively in 28 cases (8.2%). 44 additional nsPCa were detected by SB and 34 by TB (Table 4). The PCa detection according to the PI-RADS classification v2.1 is shown in Table 5. In 98% of patients in PI-RADS assessement category 5 and in 62% of patients in PI-RADS category 4 a PCa was histopathologically proven, including 85% and 44% csPCa, respectively. Therefore, a sensitivity of 94% and specificity of 54% for all PCa and 98% and 44% for csPCa was achieved (Table 6**)**. The .xls file “MRI_Biopsy_Data” contains clinical, MRI, biopsy, and histopathological variables (**Supp. document**).

**Table 1:** MRI protocols.

3T Magnetom PRISMA® (Siemens Healthcare GmbH)							
**Sequence**	T2 TSE			rs-EPI	ptx-EPI	T1 TSE	T1 Vibe DCE
**Orientation**	Sagittal	Coronal	Axial	Axial	Axial	Axial	Axial
**TR (ms)**	4060	3880	3990	5450	3500	870	3.87
**TE (ms)**	101	99	102	50/80	67	13	1.46
**Matrix size**	320	320	256	140	82	576	256
**Thickness (mm)**	3 mm	3 mm	3 mm	3 mm	3 mm	5 mm	3 mm
**Voxel size (mm)**	0.5 × 0.5 × 3	0.5 × 0.5 × 3	0.5 × 0.5 × 3	1.4 × 1.4 × 3	0.9 × 0.9 × 3	0.6 × 0.6 × 5	0.8 × 0.8 × 3.3
**FOV (mm)**	170	170	130	200	150	350	200
**b-values (sec/mm2)**	-	-	-	0, 1000 + 1800 (calculated)	50, 500, 1000 + 2000 (calculated)	-	-
**Acquisition time (min:sec)**	3:23	4:16	5:19	6:38	3:35	2:35	3:06

**3T Magnetom SKYRA®** (Siemens Healthcare GmbH)							

**Sequence**	T2 TSE			rs-EPI	ss-EPI	T1 TSE	T1 Vibe DCE

**Orientation**	Sagittal	Coronal	Axial	Axial	Axial	Axial	Axial
**TR (ms)**	7870	7430	8770	6200	7800	712	3.90
**TE (ms)**	98	98	79	56/84	88	13	1.39
**Matrix size**	320	320	256	112	136	576	256
**Thickness (mm)**	3 mm	3 mm	3 mm	3 mm	3 mm	5 mm	3 mm
**Voxel size (mm)**	0.5 × 0.5 × 3	0.5 × 0.5 × 3	0.5 × 0.5 × 3	1.8 × 1.8 × 3	1.5 × 1.5 × 3	0.6 × 0.6 × 5	0.8 × 0.8 × 3
**FOV (mm)**	170	170	130	200	200	350	200
**b-values (sec/mm2)**	-	-	-	0, 1000 + 1600 (calculated)	0, 500, 1000 + 1500 (calculated)	-	-
**Acquisition time (min:sec)**	2:45	3:06	4:58	7:33	5:08	3:11	3:02

3T Magnetom Trio® (Siemens Healthcare GmbH)						
**Sequence**	T2 TSE			ss-EPI	T1 TSE	T1 Vibe DCE
**Orientation**	Sagittal	Coronal	Axial	Axial	Axial	Axial
**TR (ms)**	11330	11330	10630	4700	650	3.62
**TE (ms)**	103	103	117	90	13	1.27
**Matrix size**	256	256	256	136	320	128
**Thickness (mm)**	3 mm	3 mm	3 mm	3 mm	5 mm	3 mm
**Voxel size (mm)**	0.7 × 0.7 × 3	0.7 × 0.7 × 3	0.5 × 0.5 × 3	1.5 × 1.5 × 3	1.3 × 0.9 × 5	1.5 × 1.5 × 3.3
**FOV (mm)**	170	170	128	200	300	192
**b-values (sec/mm2)**	-	-	-	0, 500, 1000 + 1400 (acquired)	-	-
**Acquisition time (min:sec)**	4:11	4:11	8:21	4:31	5:15	3:36

TSE = turbo spin echo; rs-EPI = readout-segmented multi-shot echo-planar imaging; ss-EPI = single-shot echo-planar imaging; ptx-EPI= parallel-transmit echo-planar imaging; TR = repetition time, TE = echo time; FOV = field of view

**Table 2:** Prostate cancer (PCa) detection by biopsy indication.

	Primary biopsy n=373	Secondary biopsy n=327	Patients on AS n=85
**All PCa** n=461	66% (245)	45% (148)	80% (68)

**csPCa** n=342	51% (190)	34% (112)	47% (40)

**Note. –** PCa = prostate cancer; csPCa = clinical significant prostate cancer; AS = active surveillance.

**Table 3:** Prostate cancer (PCa) detection rates of MRI-targeted (TB) and systematic (SB) biopsy.

n=785	All PCa	csPCa	nsPCa	No PCa
**Combined SB + TB**	59% (461)	44% (342)	15% (119)	41% (324)
**TB**	49% (387)	38% (300)	11% (87)	51% (398)
**SB**	46% (360)	32% (247)	14% (113)	54% (425)

**Note. –** PI-RADS = Prostate Imaging Reporting and Data System, version 2.1; csPCa = clinical significant prostate cancer; nsPCa = non-significant Prostate cancer; PCa = prostate cancer; SB = systematic biopsy; TB = MRI/US-fusion-guided biopsy.

**Table 4:** Cross table of prostate cancer (PCa) detection of MRI-targeted (TB) and systematic (SB) biopsy.

		SB			
		csPCa	nsPCa	No PCa	total
**TB**	**csPCa**	205	28	**67**	300
	**nsPCa**	12	41	34	87
	**No PCa**	**30**	44	324	398
	**total**	247	113	425	**785**

**Note. –** csPCa = clinical significant prostate cancer; nsPCa = non-significant prostate cancer; PCa = prostate cancer; SB = 12-core transrectal ultrasound-guided biopsy; TB = MRI/US-fusion-guided biopsy.

**Table 5:** Prostate cancer (PCa) detection rates according to the overall PI-RADS classification (v2.1) on mp-MRI per patient.

PI-RADS	Patients	All PCa	csPCa	nsPCa
**1 or 2**	3% (25)	-	-	-
**3**	23% (177)	15% (26)	3.4% (6)	11% (20)
**4**	49% (386)	62% (241)	44% (169)	19% (72)
**5**	25% (197)	98% (194)	85% (167)	14% (27)

**Note. –** PI-RADS = Prostate Imaging Reporting and Data System, version 2.1; PCa = prostate cancer; csPCa = clinically significant prostate cancer; nsPCa = non-significant prostate cancer.

**Table 6:** Accuracy of PI-RADS classification (v2.1).

	All PCa		csPCa	
PI-RADS	*value*	*95% CI*	*value*	*95% CI*
**Sensitivity**	**94%**	0.92 – 0.96	**98%**	0.96 – 0.99
**Specificity**	**54%**	0.49 – 0.60	**44%**	0.40 – 0.49
**NPV**	**87%**	0.82 – 0.91	**97%**	0.94 – 0.99
**PPV**	**75%**	0.71 – 0.78	**58%**	0.54 – 0.62

**Note. –***Positive MRI defined as ≥ PI-RADS 4.* PI-RADS = Prostate Imaging Reporting and Data System, version 2.1; PCa = prostate cancer; csPCa = clinically significant prostate cancer; NPV = negative predictive value; PPV = positive predictive value.

## 2 Experimental Design, Materials and Methods

Patients with elevated PSA who received a 3T mp-MRI of the prostate in accordance with the current PI-RADS recommendations were enrolled in this study [2,3]. Patients with subsequent combined 12-core SB and TB using elastic fusion of real-time ultrasound segmentation data by UroNAV biopsy system (Philips Healthcare, Invivo Corporation, USA) were finally retrospectively analyzed. Prostate segmentation (MRI contouring of the prostate gland boundary) and lesion registration (marking the cancer suspicious regions (mCSR) in the axial T2 images for targeted biopsy with a 3D region of interest (ROI) of the total lesion and a sub-ROI of the lesion center correlating to the area with the lowest apparent diffusion coefficient (ADC) value) were performed using the DynaCAD software (Version 3 or 4, Philips Healthcare, Invivo Corporation, USA). Two targeted cores were obtained from each registered mCSR described in the mp-MRI report. All biopsy cores from TB and SB were histopathologically evaluated in accordance with the recommendations of the International Society of Urological Pathology (ISUP). CsPCa was defined as ISUP grade group 2 or higher in at least one lesion; Gleason score ≥ 3+4=7 [4].

We retrospectively analyzed the mp-MRI data in cases with a Gleason upgrade or csPCa detection by only SB and not in TB with three radiologists in consensus [1]. Visibility of the highest Gleason score (most aggressive PCa part; index lesion; IL) in the mp-MRI and correlation with the described mCSR was done. In cases with missed csPCa by TB possible reasons (IL localization, IL diameter, MRI quality, biopsy quality) with consecutive inaccurate segmentation (MRI and/or US contouring of the prostate gland boundary) or registration (contouring of the IL, respectively of the assumable most aggressive center of the lesion) for MRI/US fusion-guided biopsy were assessed [1].

## Ethics Statement

All patients signed a written informed consent and our institutional review board approved this trial (Medical Faculty of the Heinrich-Heine-University Düsseldorf; Study-Nr: 5910R). Our study was conducted in accordance with the Declaration of Helsinki.

## CRediT Author Statement

**Maximilian Klingebiel:** Writing – original draft, Data curation, Visulization, Formal analysis; **Christian Arsov:** Investigation, Validation, Data curation, Resources, Writing – review & editing; **Tim Ullrich:** Writing – original draft, Writing – review & editing, Validation, Formal analysis, Data curation; **Michael Quentin:** Investigation, Resources, Writing – review & editing; **Rouvier Al-Monajjed:** Investigation, Resources; **David Mally:** Investigation, Resources, Data curation; **Lino Morris Sawicki:** Investigation, Resources, Writing – review & editing; **Andreas Hiester:** Investigation, Resources, Data curation; **Irene Esposito:** Investigation, Validation, Resources, Data curation, Supervision, Writing – review & editing; **Peter Albers:** Resources, Validation, Supervision, Writing – review & editing; **Gerald Antoch:** Supervision, Writing – review & editing; **Lars Schimmöller:** Conceptualization, Methodology, Validation, Supervision, Project administration, Formal analysis, Data curation, Visulization, Writing – original draft, Writing – review & editing

## Declaration of Competing Interest

The authors declare that they have no known competing financial interests or personal relationships which have or could be perceived to have influenced the work reported in this article.

## Data Availability

MRI_Biopsy_Data.xls (Original Data) (Zenodo).

## Appendix Supplementary materials

Image, application 1
